# Exercise training does not improve myocardial diastolic tissue velocities in Type 2 diabetes

**DOI:** 10.1186/1476-7120-5-32

**Published:** 2007-09-26

**Authors:** Antti Loimaala, Kaj Groundstroem, Marjo Rinne, Arja Nenonen, Heini Huhtala, Ilkka Vuori

**Affiliations:** 1 Clinical Physiology and Nuclear Medicine, Seinäjoki Central Hospital, Seinäjoki, Finland; 2 Department of Medicine, Kymenlaakso Central Hospital, Kotka, Finland; 3 UKK institute for Health Promotion Research, Tampere, Finland; 4 Clinical Chemistry, Rheumatism Foundation Hospital, Heinola, Finland; 5 School of Public Health, University of Tampere, Finland

## Abstract

**Background:**

Myocardial diastolic tissue velocities are reduced already in newly onset Type 2 diabetes mellitus (T2D). Poor disease control may lead to left ventricular (LV) systolic dysfunction and heart failure. The aim of this study was to assess the effects of exercise training on myocardial diastolic function in T2D patients without ischemic heart disease.

**Methods:**

48 men (52.3 ± 5.6 yrs) with T2D were randomized to supervised training four times a week and standard therapy (E), or standard treatment alone (C) for 12 months. Glycated hemoglobin (HbA_1c_), oxygen consumption (VO_2max_), and muscle strength (Sit-up) were measured. Tissue Doppler Imaging (TDI) was used to determine the average maximal mitral annular early (Ea) and late (Aa) diastolic as well as systolic (Sa) velocities, systolic strain (***ε***) and strain rate (***έ***) from the septum, and an estimation of left ventricular end diastolic pressure (E/Ea).

**Results:**

Exercise capacity (VO_2max_, E 32.0 to 34.7 vs. C 32.6 to 31.5 ml/kg/min, p = .001), muscle strength (E 12.7 to 18.3 times vs. C 14.6 to 14.7 times, p < .001), and HbA_1c _(E 8.2 to 7.5% vs. C 8.0 to 8.4%, p = .006) improved significantly in the exercise group compared to the controls (ANOVA). Systolic blood pressure decreased in the E group (E 144 to 138 mmHg vs. C 146 to 144 mmHg, p = .04). Contrary to risk factor changes diastolic long axis relaxation did not improve significantly, early diastolic velocity Ea from 8.1 to 7.9 cm/s for the E group vs. C 7.4 to 7.8 cm/s (p = .85, ANOVA). Likewise, after 12 months the mitral annular systolic velocity, systolic strain and strain rate, as well as E/Ea were unchanged.

**Conclusion:**

Exercise training improves endurance and muscle fitness in T2D, resulting in better glycemic control and reduced blood pressure. However, myocardial diastolic tissue velocities did not change significantly. Our data suggest that a much longer exercise intervention may be needed in order to reverse diastolic impairment in diabetics, if at all possible.

## Background

Type 2 diabetes mellitus (T2D) is a strong risk factor for coronary heart disease and heart failure [1,2]. Patients with poor glycemic control and hypertension have a worse prognosis [3,4], which may at least partially attribute to myocardial disease. It has been shown previously that although left ventricular (LV) ejection fraction is normal, myocardial diastolic tissue velocities are reduced already in the early phase of the disease, indicating incipient diabetic heart muscle disease [5]. When the disease progresses also systolic dysfunction with wall motion abnormalities occur [6,7]. More interestingly, increased left ventricular filling pressure associates with higher mortality after myocardial infarction in males, which underlines the importance of effective treatment of patients at risk [8]. However, to date there is no established treatment to reverse diastolic dysfunction in T2D. It has been shown that good glucose control decreases the incidence of vascular complications [9], and lipid lowering therapy reverses myocardial hypertrophy and increases tissue velocities [10]. We thus hypothesized that enhanced myocardial function after a physical exercise program might also be observed. Tissue Doppler Imaging (TDI) gives accurate velocity measures from individual myocardial segments in various populations and disease states, and is suited for monitoring sub-clinical changes during interventions [11]. The aim of this study was to investigate the effects of a randomized, controlled long-term exercise-training program on major risk factors of T2D, and as primary end-point measures on myocardial early diastolic tissue velocities in middle-aged diabetic patients without clinical macro- or micro vascular complications.

## Methods

The study group consisted of 48 men with T2D diagnosed less than three years earlier. Exclusion criteria were history or ECG signs of myocardial infarction, conduction disturbance on ECG or history of arrhythmias, symptoms or ECG signs of ischemia on maximal treadmill stress test, wall motion abnormalities on resting echocardiogram, significant valvular disease, any lung disease, history of cardiomyopathy, rheumatoid arthritis or connective tissue disease, insulin treatment, or any chronic disease other than diabetes. Altogether 33 patients were treated with oral hypoglycemic drugs and 20 patients were treated for hypertension (ACE inhibitors 10, β blockers 2, Ca antagonist 3, and ACE inhibitor combined with a diuretic or Ca antagonist 5 patients).

After a detailed physical examination, the subjects were randomized as follows: 1) control group (C), standard treatment of T2D; 2) exercise group (E), standard treatment of T2D plus jogging or walking twice a week at a heart rate level corresponding to 65–75% of maximal oxygen consumption (VO_2max_) on spiroergometry, and muscle strength training twice a week at a predefined intensity. The resistance training program was progressive, every session included eight exercises for large muscle groups alternating between trunk and upper and lower extremities. Resistance for each exercise station was assessed by repeated maximum (RM) tests. Three sets of 10–12 repetitions at 70–80% maximum voluntary contraction were performed. Exercises in the training program were changed every two months, and a new target heart rate was set every six months based on changes in VO_2max _measured during a follow-up treadmill test. Two sessions out of four per week were supervised by a physiotherapist, and heart rate and intensity controlled (Polar Smart Edge, Polar Electro Oy, Kempele, Finland) and all sessions were recorded in a diary. The minimum duration of a session was 30 minutes at the target heart rate or intensity, and the intervention lasted for 12 months. The investigation conforms with the principles outlined in the declaration of Helsinki [12]. The study was approved by the Research Ethics Committee of the Tampere University Hospital, and all subjects signed an informed consent.

### Measurements

All subjects performed a maximal treadmill exercise stress test according to a standard protocol. Fasting (12 h) blood samples were taken, and blood glucose was assessed by the glucose dehydrogenase method (mmol/l), and glycated hemoglobin (HbA_1c_, %) was assessed by the immunoturbidimetric method (Roche Ltd, Basel, Switzerland). Training and vigorous exercise were not allowed for 24 hours before blood was drawn and 12-hour restriction was applied for tea and caffeine products. Cobas Mira Plus and Cobas Integra automatic analyzers were used for the analyses. Total cholesterol was measured from serum and lipoprotein fractions were determined using an enzymatic method (CHOD-PAP, Boehringer Mannheim, Mannheim, Germany). High density lipoprotein (HDL) subfractions were determined according to Kirstein and Carlson [13]. Triglycerides were measured from frozen samples by enzymatic hydrolysis (GPO-PAP, Boehringer Mannheim, Mannheim, Germany). LDL cholesterol level was calculated using Friedewald's formula [14].

#### Echocardiographic and tissue Doppler myocardial imaging measurements

2-D and M-mode echocardiographic techniques and projections were used in a standard fashion [15]. All images were stored on magneto-optic discs using a commercially available echocardiograph (VingMed System V, Horten, Norway). Measurements were performed off-line. The physician performing the measurements was unaware of the clinical and study group data. Standard left ventricular dimensions were taken from three consecutive cardiac cycles. Color-coded TDI cine loops from the apical 4- and 2-chamber views with > 100 frames per second were used for TDI measurements. Mean peak systolic (Sa, cm/s), and early (Ea, cm/s) and late (Aa, cm/s) diastolic tissue velocities from the lateral mitral annulus were measured. Additionally, peak systolic strain (***ε***, %) and systolic strain rate (***έ***, 1/s) were measured from the septum in order to assess local myocardial deformation. As a non-invasive estimate of the left ventricular end-diastolic pressure, the ratio of mitral inflow early diastolic velocity to mitral annular early diastolic velocity was determined (E/Ea). An average of at least three measurements for the individual variables was used in statistical analyses. Normal TDI values for middle-aged men determined in our laboratory are: Sa 7.8 ± 2.6 cm/s (p = .14 vs. T2D) and Ea 10.4 ± 2.6 cm/s (p = .001 vs. T2D), Aa 4.8 ± 1.2 (p = .23 vs. T2D).

### Statistical methods

Clinical characteristics of the intervention group and controls are presented as means and standard deviations (SD). Comparisons of clinical study variables between the study groups were performed at baseline by analysis of covariance with age as a covariate. Detection of a clinically significant effect of exercise training on tissue velocities, i.e. an increase of at least 20% in Ea velocity and a standard deviation of the estimate of 0.7 cm/s, with 80% power and at the 5% significance level, would require 17 patients in both study arms at study end. In order to compare the systolic and diastolic velocities between the exercise and the control group after the intervention, analysis of covariance with baseline velocities and heart rate during TDI measurements was used as a covariate, and the results are presented as means, standard errors and 95% confidence intervals for the differences (CI) (SPSS 12.0.1).

## Results

Clinical and standard echocardiographic characteristics of the subjects are presented in Table 1. Significant differences between the study groups were not observed at baseline except that the LV internal diameter in diastole was higher in the controls and the posterior wall thicker in the E group. Marked changes in medication were not made during the intervention, and use of sulfonylureas or metformin/insulin sensitizers was stable, two subjects in the C group started insulin treatment. The left ventricular ejection fraction was similar before and after the intervention. Table 2 summarizes the follow-up data on exercise performance, blood pressure, mitral inflow velocities and blood analyses. The E group met the requirements for duration and intensity of both the endurance and muscle strength training programs, average adherence to controlled sessions was 1.5 times of two. VO_2max _improved in the E group (Exercise +8.0%, Control -3.3%), and so did muscle strength (Exercise +44%, Control 0%, Table 2). A significant decrease in HbA_1c _occurred in the E group, whereas glucose control slightly worsened in the controls. Plasma insulin was higher at entry and it decreased more during the intervention in the E group, but the difference was not statistically significant. SBP decreased on average by 6 mmHg in the intervention group and by 2 mmHg in the controls, the difference being significant (Table 2). Plasma lipids at entry were quite similar in both groups and marginally above current recommendations for diabetic patients. During the intervention, only minor changes were observed, but the net differences in total and HDL cholesterol were nearly significant (Table 2). Mitral inflow velocities at the beginning and at study end were not significantly different between the study groups.

**Table 1:** Clinical and echocardiographic characteristics of    the study subjects at baseline. Values are means (SD).

Group	Control (n = 24)	Exercise (n = 24)	p value
Variable			
Age (years)	52.8 (6.0)	52.8 (5.2)	0.98
Weight (kg)	94.1 (12.8)	90.2 (9.4)	0.22
SBP (mmHg)	146 (15)	144 (17)	0.60
DBP (mmHg)	89 (6)	88 (9)	0.77
EF (%)	68 (7)	71 (9)	0.32
IVSD (mm)	10 (2)	10 (2)	0.27
LVEDD (mm)	55 (6)	49 (7)	0.003
PWD (mm)	10 (2)	11 (1)	0.04

SBP = systolic blood pressure; DBP = diastolic blood pressure; EF = left ventricular ejection fraction; IVSD = inter-ventricular septal thickness in diastole; LVEDD = left ventricular internal diameter in diastole; PWD = posterior wall thickness in diastole.

**Table 2:** Exercise and clinical data on the study groups. Values are means (SE).

Group		Control (n = 24)	Exercise (n = 24)	p value
Variable				
VO_2max_	0 mo	32.6 (1.1)	32.0 (1.1)	
(ml/kg/min)	12 mo	31.5 (0.6)	34.7 (0.6)	
	Net diff		3.2	
	95% CI		1.4 to 5.0	0.001
Weight	0 mo	94.1 (2.2)	90.0 2.2)	
(kg)	12 mo	92.4 (0.7)	91.4 (0.7)	
	Net diff		-1.4	
	95% CI		-2.8 to 0.1	0.072
Sit-up	0 mo	14.6 (9.6)	12.7 (7.3)	
(times)	12 mo	14.7 (1.2)	18.3 (1.2)	
	Net diff		6.7	
	95% CI		4.3 to 9.0	< 0.001
HBA1c	0 mo	8.0 (1.3)	8.2 (2.1)	
(%)	12 mo	8.4 (0.2)	7.5 (0.2)	
	Net diff		-0.9	
	95% CI		-1.6 to -0.3	0.006
Insulin	0 mo	13.6 (2.3)	16.7 (2.3)	
(mmol/l)	12 mo	13.6 (1.3)	11.7 (1.3)	
	Net diff		-1.9	
	95% CI		-5.6 to 1.8	0.31
Cholesterol	0 mo	4.85 (.18)	4.63 (.18)	
(mmol/l)	12 mo	4.89 (.11)	4.61 (.11)	
	Net diff		-0.28	
	95% CI		-.58 to 0.03	0.07
HDL	0 mo	1.13 (.05)	1.09 (.05)	
(mmol/l)	12 mo	1.18 (.03)	1.12 (.03)	
	Net diff		-0.06	
	95% CI		-.13 to .02	0.14
LDL	0 mo	3.24 (.17)	3.15 (.17)	
(mmol/l)	12 mo	3.17 (.10)	3.10 (.10)	
	Net diff		-0.7	
	95% CI		-.35 to .22	0.68
Triglycerides	0 mo	1.83 (.15)	1.70 (.15)	
(mmol/l)	12 mo	1.80 (.13)	1.70 (.13)	
	Net diff		-0.09	
	95% CI		-.45 to 0.26	0.60
SBP	0 mo	146 (15)	144 (17)	
(mmHg)	12 mo	144 (1.9)	138 (1.9)	
	Net diff		-5.5	
	95% CI		-10.8 to 0.2	0.041
E (m/s)	0 mo	0.69 (.03)	0.64 (.03)	
	12 mo	0.70 (.03)	0.71 (.03)	
	Net diff		.01	
	95% CI		-.07 to .09	0.77
A (m/s)	0 mo	0.66 (.03)	0.68 (.03)	
	12 mo	0.69 (.02)	0.71 (.02)	
	Net diff		.02	
	95% CI		-.04 to .09	0.46

VO_2max _= maximal oxygen consumption; 0 mo/12 mo = measurements at baseline and after 12 months of intervention; Net diff. = difference of change compared to the control group (analysis of covariance); 95% CI = 95% confidence interval for the net difference; Weight = body weight; Sit-up = sit-up test, number of repetitions; HBA1c = glycated hemoglobin; Insulin = serum insulin; Cholesterol = total cholesterol; HDL = high-density cholesterol; LDL = low-density cholestrol; Triglycerides = blood triglyceride concentration; SBP = systolic blood pressure; E = mitral inflow early diastolic velocity; A = mitral inflow late diastolic velocity.

**Figure 1:**
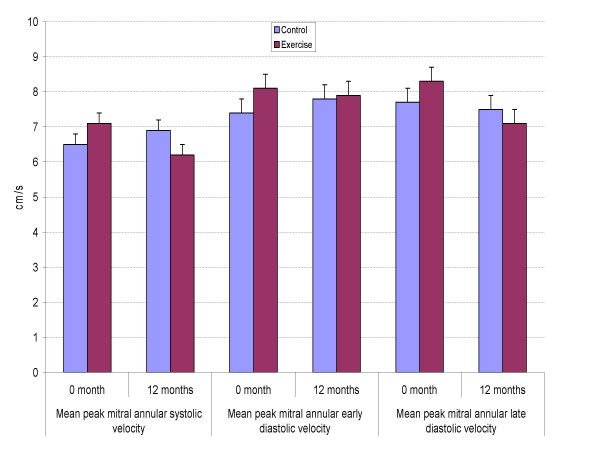
Mean peak mitral annular long-axis tissue doppler velocities (cm/s) in the study groups at baseline and after 12 months. Values are means (SE).

**Figure 2:**
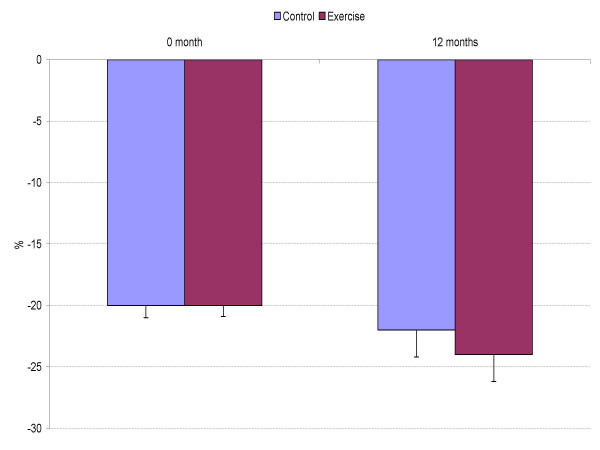
Maximal systolic strain (%) from the septum.

**Figure 3:**
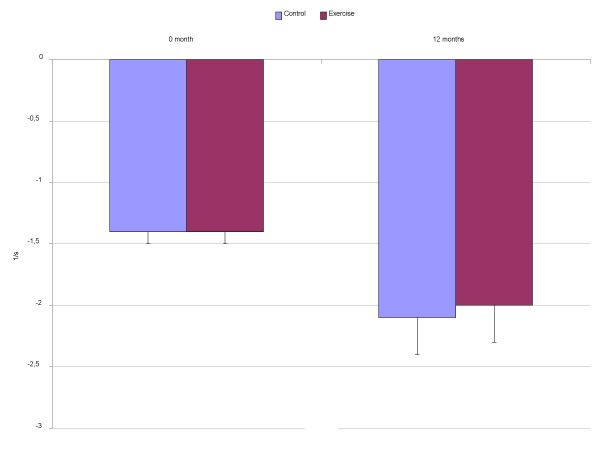
Maximal systolic strain rate (1/s) from the septum.

Tissue Doppler measurements at baseline and at follow-up are presented in Figures 1 to 3. The average maximal mitral annular tissue velocities were slightly lower in the C group and unaffected by anti-hypertensive medication at baseline. During the 12 months only non-significant changes in the mitral annular early and late diastolic velocities were observed and they are within measurement variability (Figure 1). Likewise, systolic velocity did not improve in the E group. When myocardial deformation was evaluated from the septum, changes in strain and strain rate (Figures 2 and 3) were negligible, average maximal systolic strain improved slightly, being -24% at study end for the E group vs. -22% for the C group. However, baseline values were normal. At study entry, mean E/Ea was 8.4 in the E group and 9.4 in the C group (p = 0.29 adjusted for age, ANOVA), the values being near normal. Eleven subjects in the C group and 10 in the E group had a ratio above 8 but again, significant differences after the intervention were not observed for E/Ea (E 9.0 vs. C 8.5, p = 0.42).

## Discussion

Lower myocardial tissue velocities and increased myocardial reflectivity in diabetes indicate altered myocardial structure and diastolic dysfunction is a dominant feature [6,16]. To our knowledge, this is the first controlled study examining the effects of a long-term, high intensity exercise program on myocardial long-axis relaxation in T2D. Patients with hypertrophic cardiomyopathy (HCMP) without established myocardial hypertrophy have significantly lower myocardial systolic tissue velocities compared to normal subjects [17]. The lower early diastolic resting velocities (25% on average compared to healthy controls) in our population suggest a specific diabetic heart muscle disease, predominantly diastolic dysfunction. It is characterized by several abnormalities such as replacement fibrosis as well as decreased myocardial capillary density, altered sarcoplasmic reticular calcium transport, accumulation of fatty acid metabolites in myocytes, and changes in autonomic innervation of the heart [2]. The primary aim of our study was to examine whether multifactorial risk factor reduction can reverse myocardial diastolic impairment or not. Although risk factors were reduced significantly and the glucose control improved, these changes did not translate into effect on measured long axis tissue velocities or deformation. In fact, the observed changes are in the range of method variability. It seems that reversal of the functional changes in the human diabetic heart requires a much longer exercise intervention.

Patients with T2D have higher cardiovascular morbidity compared to healthy subjects [18]. Diabetic heart disease may develop and be a cause for excessive morbidity and hospitalization for heart failure in long-lasting disease. The study patients represent an average T2D population; they were overweight and hypertensive with medication (45%), and their glucose control was fair-to-poor. None of the patients had a history or clinical evidence of micro vascular complications or coronary artery disease on treadmill exercise stress test. It is well known that physical exercise reduces mortality from cardiovascular causes in several populations [19], e.g. by reducing blood pressure, changing the lipid profile, and also perhaps by enhancing the autonomic control of the heart [20]. Our patients reached a significantly better glucose balance than the controls, which is comparable to the results of intensive medical treatment [9]. In addition, their systolic blood pressure decreased and VO_2max _improved significantly, i.e. the training was effective.

Sylvén et al. [21] reported that objective improvement in myocardial systolic function and frequency of angina episodes can be obtained by gene therapy in inoperable CAD. Along with new collateral vessel formation, myocardial perfusion improved and this resulted in 25% higher myocardial systolic tissue velocities during dobutamine stress. If ischemia is the limiting factor of function, better oxygen supply to the myocardium increases systolic tissue velocities at stress. Our patients achieved higher oxygen consumption after the intervention and thus their cardiac output at peak stress was improved. However, our patients had lower *resting *long-axis diastolic velocities at baseline compared to healthy subjects and no improvement occurred. Diastolic velocities are dependent on the elastic properties of the left ventricle that are greatly influenced by increased collagen deposition and other aforementioned factors [2,16]. The endocardial myocytes suffer earlier from sub-clinical ischemia than circular epicardial fibers, which may result in lower systolic and diastolic long-axis velocities. If true reversal of abnormalities had occurred, an increase in diastolic velocities of at least 20% would have been enough in our study. We have shown recently that exercise training improves baroreflex sensitivity in diabetics [22], which most likely is a result of enhanced endothelial function. If so, the lower resting diastolic tissue velocities in our patients are not due to poor microcirculation and endocardial ischemia solely [7], but to structural changes which are more resistant to conventional therapy (or irreversible). It thus seems that "the tissue velocity gap" of about 25% to normal early diastolic tissue velocities needs a much longer or even more intensive exercise program [5].

According to Patel et al., myocardial hypertrophy can be reversed and myocardial function improved by simvastatin in animals [10]. Concomitantly with the reduction of the LV dimensions, the collagen volume fraction was reduced and these resulted in significantly higher, 30% on average, systolic and diastolic septal velocities in the treated rats compared to the placebo group, and velocities at the end of the study were near normal. More importantly, Weidemann et al. demonstrated in 16 patients with Fabry disease that giving agalsidase β for twelve months improves myocardial systolic function, both strain rate (from 2.8/s to 3.7/s) and strain (from 34 % to 45%) improved significantly and myocardial mass was reduced [23]. In our patients, significant changes in LV dimensions were not observed nor expected, although long-standing physical training induces LV hypertrophy. Nevertheless, improved metabolic control, decreased afterload, and sympathetic nerve activity due to lower blood glucose levels could have beneficial effects on myocardial diastolic function. It seems that significant changes in major risk factors by exercise do not translate into enhanced myocardial relaxation during one-year follow-up unlike the structural changes produced by agressive medical therapy [10,23]. Our results concur with those observed for a one-month medical therapy in T2D patients [24]. These findings suggest that the insignificant changes in tissue velocities and also in myocardial deformation are due to a too short training period, or that the mechanisms activated by medical therapy are different and more effective. Although tissue velocities measured during stress might be a more sensitive indicator of effect, our study design was analogous to the Fabry study where resting velocities were measured as well, and a clear treatment effect was observed.

### Clinical implications

Morbidity and costs for T2D increase rapidly worldwide [25]. This is mainly due to changes in dietary habits and lack of exercise, which results in an enormous increase in diabetes-related complications. To date there is no known treatment to reverse diabetic heart disease. This study shows that, in order to repair the end-organ damage produced by decades of sedentary western life style, a much longer non-pharmacological intervention (or aggressive medical therapy) is needed to obtain improvement in myocardial diastolic function in T2D. As demonstrated recently, prevention of T2D conservatively is effective and should be the first-line regimen to reduce the incidence of diabetic complications [26]. In subjects with established disease, good glycemic and risk factor control may slow down the process of diastolic dysfunction, and postpone the development of cardiac failure [27,28]. However, if diastolic dysfunction is already present, the functional "reserve" is smaller after myocardial infarction compared to patients with normal hearts, and the patient outcome is unfavorable.

A limitation of the study is that, although the change in systolic blood pressure was statistically significant, it was still quite moderate and smaller than by medical therapy in general [29]. Food diaries were not used and consumption of saturated fats was not known. However, dietary advice is always given to T2D patients by GPs and the lipid levels were moderate in both study groups, and more importantly, this was an exercise intervention. Significant weight reduction would have enhanced the blood pressure decrease, which did not occur. Most obviously the muscle mass replaced body fat mass. Despite these limitations, the metabolic control was improved significantly together with enhanced autonomous control of the heart. However, further and much longer randomized studies are needed to clarify the effect of physical exercise on myocardial function in diabetes.

## Competing interests

The author(s) declare that they have no competing interests.

## Authors' contributions

AL and KG carried out study design, echocardiography imaging and measurements, data analyses and reporting. MR designed and supervised all exercise programs, and critically revised the manuscript. AN designed and performed all blood analyses and revised the manuscipt. HH designed and performed statistical analyses together with AL, and critically revised the manuscript. IV caried out study design and exercise program planning, and revised the manuscript critically. All authors read and approved the final manuscript.
